# *Mycobacterium abscessus* persistence in the face of *Pseudomonas aeruginosa* antagonism

**DOI:** 10.3389/fcimb.2025.1569331

**Published:** 2025-05-09

**Authors:** Rashmi Gupta, Martin Schuster, Kyle H. Rohde

**Affiliations:** ^1^ Division of Immunity and Pathogenesis, Burnett School of Biomedical Sciences, College of Medicine, University of Central Florida, Orlando, FL, United States; ^2^ Division of Microbiology, College of Science, Oregon State University, Corvallis, OR, United States

**Keywords:** *Mycobacterium abscessus*, *Pseudomonas aeruginosa*, mixed microbial interactions, dual RNAseq, secreted factors, quorum sensing

## Abstract

**Introduction:**

Chronic bacterial infections are responsible for significant morbidity and mortality in cystic fibrosis (CF) patients. *Pseudomonas aeruginosa* (*Pa*), the dominant CF pathogen, and *Mycobacterium abscessus* (*Mab*) can individually cause persistent, difficult to treat pulmonary infections. Co-infection by both pathogens leads to severe disease and poor clinical outcomes. Although interactions between *Pa* and other co-infecting pathogens in CF patients have been the focus of numerous studies, the dynamics of *Pa-Mab* interactions remain poorly understood.

**Methods:**

To address this knowledge gap, the study examined how *Mab* and *Pa* influenced each other through culture-based growth assays and molecular-based dual RNAseq analysis. Growth was measured by CFU determination and luminescence reporter -based readouts.

**Results:**

In initial studies, we noted that the growth of *Pa* continued unimpeded in a planktonic co-culture model, whereas *Pa* appeared to exert a bacteriostatic effect on *Mab*. Strikingly, exposure of *Mab* to cell-free spent supernatant of *Pa* resulted in a dramatic, dose-dependent bactericidal effect. Initial characterization indicated that this potent *Pa*-derived anti-*Mab* cidal activity was mediated by a heat-sensitive, protease-insensitive soluble factor of >3kDa size. Further analysis demonstrated that expression of this mycobactericidal factor requires LasR, a central regulator of *Pa* quorum sensing (QS). In contrast, ΔLasR *Pa* was still able to exert a bacteriostatic effect on Mab during co-culture, pointing to additional LasR-independent factors able to antagonize *Mab* growth. However, the ability of *Mab* to adapt during co-culture to counter the cidal effects of a LasR regulated factor suggested complex interspecies dynamics. Dual RNAseq analysis of *Mab*-*Pa* co-cultures revealed significant transcriptional remodeling of *Mab*, with differential expression of 68% of *Mab* genes compared to minimal transcriptional changes in *Pa*. Transcriptome analysis reflected slowed *Mab* growth and metabolic changes akin to a non-replicating persister phase. A tailored *Mab* response to *Pa* was evident by enhanced transcript levels of genes predicted to counteract alkylquinolone QS signals, respiratory toxins, and hydrogen cyanide.

**Discussion:**

The study showed *Mab* is capable of coexisting with *Pa* despite *Pa*’s antagonistic effects, eliciting an adaptive molecular response in *Mab*. This study provides the first transcriptome-level insight into genetic interactions between the two CF pathogens offering potential strategies for disrupting their communities in a CF lung to improve patient clinical performance. Moreover, identification of a novel antimicrobial natural product with potent cidal activity against *Mab* could lead to new drug targets and therapies for Mab infections.

## 1 Introduction

Chronic microbial infections are responsible for morbidity and mortality in patients suffering with lung disorders, in particular cystic fibrosis (CF). CF is an autosomal recessive disorder characterized by a defect in cystic fibrosis transmembrane conductance regulator (CFTR) that regulates chloride and bicarbonate secretion across the pulmonary epithelium (Poulsen et al., 1994; Mall and Hartl, 2014; Elborn, 2016; Kunzelmann et al., 2017). This results in improper pH and salt balance leading to accumulation of thick, sticky mucus and defective mucociliary clearance from lung airways. This environment in which innate immune mechanisms are impaired is conducive for opportunistic pathogens to thrive, causing lung deterioration and poor quality of life of CF patients. Among the diverse CF microbial community, notable bacterial pathogens include *Pseudomonas aeruginosa (Pa), Staphylococcus aureus, Burkholderia cenocepacia*, and nontuberculous mycobacteria (NTM) such as *Mycobacterium abscessus* (*Mab*) (Valenza et al., 2008; Thornton and Parkins, 2023).

*Pa* is a multidrug resistant bacterial pathogen that can colonize the lungs of CF patients as early as the first few years of life (Sader et al., 2018; Recio et al., 2020). Over time, it persists and often becomes the predominant pathogen, outcompeting other colonizers in adulthood (Aebi et al., 1995; Van Daele et al., 2005; Smith et al., 2006; Pittman et al., 2011; Coburn et al., 2015; Bhagirath et al., 2016; Banjar et al., 2022; Thornton and Parkins, 2023). *Pa* produces several quorum sensing (QS) regulated virulence factors including proteases and secondary metabolites such as phenazines and hydrogen cyanide (HCN) detectable in CF sputum, which facilitate colonization of CF airways (Singh PK et al., 2000; Chambers et al., 2005; Jan, 2017). These QS mediated factors help *Pa* gain dominance within the CF microbial community by restricting competition. The QS regulatory network of *Pa* is complex and comprised of four interconnected systems - *las*, *rhl*, *pqs* and *iqs* (Latifi et al., 1996; Pesci et al., 1997; Wade et al., 2005; Lee et al., 2013). The first two systems are based on acylhomoserine lactone (AHL) signals, while the *pqs* system requires quinolone signals, and the fourth *iqs* responds to 2-(2-hydroxyphenyl)-thiazole-4-carbaldehyde signal molecules. The *las* system occupies the top position of the hierarchical QS system and is comprised of AHL signal synthase, LasI, and transcriptional regulator, LasR. This system positively regulates other systems of the QS circuitry; however these downstream pathways can also function independently of LasR under certain conditions (Latifi et al., 1996; Pesci et al., 1997; Diggle et al., 2003; Medina et al., 2003; Dekimpe and Deziel, 2009). LasR controls expression of numerous virulence genes and plays a crucial role in pathogenesis (Latifi et al., 1996; Pesci et al., 1997). Despite extensive study, the role of QS in CF infections remains perplexing. On one hand, *las* QS-regulated production of redox-active phenazine pigment pyocyanin causes pulmonary exacerbation and lung damage (Lau et al., 2004; Caldwell et al., 2009; Hunter et al., 2012; Rada and Leto, 2013). On the other hand, QS deficient LasR mutants are commonly isolated from CF patients and are responsible for enhanced neutrophil inflammation and disease progression (Smith et al., 2006; Hoffman et al., 2009; Feltner et al., 2016; Hennemann et al., 2021).

*Mab*, a second bacterial inhabitant of CF airways, is a major threat due to its increased prevalence and a high level of inherent drug resistance (Olivier et al., 2003; Sermet-Gaudelus et al., 2003; Jonsson et al., 2007; Kendall and Winthrop, 2013; Aiello et al., 2018; Thornton and Parkins, 2023; Hu et al., 2024). The prevalence of *Mab* varies with age, with reports of isolation from pediatric CF patients, but a higher prevalence in older patients over 15 years old (Pierre-Audigier et al., 2005; Roux et al., 2009). *Mab* exhibits two different colony phenotypes - smooth and rough - and both are commonly isolated from CF patients (Byrd and Lyons, 1999; Howard et al., 2006; Catherinot et al., 2009). Both phenotypes form biofilms, have similar antibiotic sensitivity and macrophage survival though the rough morphotype is considered more virulent (Greendyke and Byrd, 2008; Catherinot et al., 2009; Roux et al., 2016). It is speculated that after initial colonization of airways by smooth strains, a transition to rough phenotype occurs, leading to increased lung lesions and poor clinical outcomes (Howard et al., 2006; Catherinot et al., 2009; Kreutzfeldt et al., 2013; Hedin et al., 2023). Thus, as noted previously for *Pa*, host-derived selective pressures encountered during prolonged chronic infections may drive *Mab* evolution, altering *Mab* interactions with the host as well as with other microbes within the same niche.

There are several lines of evidence that support the relevance of *Pa-Mab* interspecies interactions. In addition to sharing environmental reservoirs in soil and water (Mangione et al., 2001), within the human host *Pa* and *Mab* both occupy the microaerobic respiratory zone in the lung (Bjarnsholt et al., 2009; Qvist et al., 2015) and are isolated from CF sputum samples (De Boeck et al., 2000; Jonsson et al., 2007; Bragonzi et al., 2012). Genomic studies that identified numerous genes shared between these species, attributed to putative horizontal gene transfer (HGT), is further evidence of interactions between *Pa* and *Mab* within the lung (Bjarnsholt et al., 2009; Ripoll et al., 2009; Qvist et al., 2015). Highlighting the clinical relevance of interspecies interactions, co-infection by these two opportunistic pathogens is known to increase disease severity and exacerbate the decline in lung function (Adjemian et al., 2018; Hsieh et al., 2018; Takano et al., 2021; Nichols et al., 2023). Yet significant knowledge gaps remain regarding the nature of *Mab-Pa* interspecies interactions and potential impact on pathogenesis (Birmes et al., 2017; Rodriguez-Sevilla et al., 2018; Rodriguez-Sevilla et al., 2019; Idosa et al., 2022). Birmes et al. demonstrated quenching of quinolone QS signals of *Pa* by *Mab* when provided exogenously to *Mab* cultures (Birmes et al., 2017). Two other research groups examined the coexistence of the two pathogens in an *in vitro* dual-species biofilm model where *Pa* exerted a negative effect on *Mab* (Rodriguez-Sevilla et al., 2019; Idosa et al., 2022). However, *Pa-*mediated inhibition of *Mab* was reported to be contact-dependent and restricted to the solid surface biofilm model, with no inhibition in the planktonic co-cultures (Idosa et al., 2022). Extensive analysis of *Pa* mutants lacking known virulence regulators (e.g. *las, rhl, pqs* QS systems), secretion systems, motility factors, and iron sequestration genes failed to identify the molecular mechanism responsible for this antagonism (Idosa et al., 2022). It was also noted that targeted killing of *Pa* with selective antibiotics favored *Mab* establishment in the dual-species biofilm (Rodriguez-Sevilla et al., 2019). It is crucial to interrogate interactions between these two human CF pathogens for a better understanding of their adaptive responses and impact on disease progression.

This study examined how *Mab* and *Pa* influenced each other through culture-based growth assays and molecular-based dual RNAseq analysis. This dual-species interaction study showed that *Mab* can persist when cultured alongside *Pa* but experiences substantial loss of viability when cultured in a cell-free *Pa* extract containing QS-dependent secreted factors. The non-cidal outcome in the co-culture and drastic changes at a molecular level in *Mab* strongly suggest that *Mab* employs adaptive mechanisms for survival and coexistence with *Pa*.

## 2 Materials and methods

### 2.1 Bacterial strains and reagents

The bacterial strains *Pa* WT (PAO1 Ochsner) (Holloway et al., 1979), Δ*lasR* (in PAO1 Ochsner background) (Wilder et al., 2011), and smooth morphotype *Mab* 390S (Howard and Byrd, 2000; Greendyke and Byrd, 2008) and *Mab-lux* (Gupta et al., 2017) were used in this study. For routine culturing and maintenance, Luria Bertani (LB) was used for both *Pa* and *Mab* strains. For selection of *Mab* for species-specific CFU enumeration from co-cultures, LB agar supplemented with 200U/ml of antibiotic polymyxin B sulfate salt (Millipore sigma) was used. No selective media was necessary for *Pa* as it forms colonies after 24 h unlike *Mab*, which takes 3–5 days.

### 2.2 *In vitro* co-culturing

A planktonic co-culture assay was used to investigate direct interactions between *Pa* and *Mab* 390S. LB-Tween 80 (0.05%) media was inoculated from a freshly streaked single colony of *Pa* strains (WT and the *lasR* mutant) or *Mab* culture started from a freezer stock. The bacterial cultures were appropriately diluted to set-up mono and co-cultures in 10 ml LB-Tween in T25 flasks. The starting OD of *Pa* and *Mab* was set at 0.05 and 0.15, respectively as used previously to account for growth rate differences (Costa et al., 2015; Birmes et al., 2017). Three monocultures: WT (P), Δ*lasR* (L), and *Mab* (MB) and the two co-cultures - *Mab*+WT *Pa* (PMB), *Mab*+Δ*lasR* (LMB) were incubated at 37°C with shaking at 150 r.p.m. for 24 h. After 24 h, 10-fold serial dilutions were prepared in PBS media and spot-plated (5 µl) onto LB agar and LB agar supplemented with polymyxin B (200U/ml) for growth of *Pa* and *Mab*, respectively for colony enumeration.

### 2.3 *Mab* growth in *Pa* supernatant

*Mab* growth was assessed in spent supernatants of WT (P) and the isogenic Δ*lasR* (L) strains of *Pa* to examine the effect of *Pa* secreted effectors. The *Pa* strains were grown overnight in LB-Tween media at 37°C, 150 r.p.m. shaking from a freshly streaked isolated colony. The cultures were diluted to OD_600_ of 0.05 and grown for 24 h. Thereafter, cultures were centrifuged (4000 r.p.m., 30 min., 4°C) and filtered through 0.22 µm pore size cellulose acetate filters (Corning, 43020). The filter-sterilized supernatant was plated (100 µl) onto LB agar plates to ensure that no *Pa* cells were present in the filtrate. *Mab* cultures were set at an OD_600_ of 0.15 in 10 ml LB-Tween (T25 flasks) with 0%, 5%, 25%, and 50% v/v, of *Pa* spent monoculture supernatant. The cultures were incubated at 37°C with shaking (150 r.p.m.) and after 0, 24, and 48 h 10-fold serial dilutions were spot-plated (5µl) onto LB agar for each time point. The CFU/ml data is an average from three biological experiments each with 4 technical replicates. The same assay was performed with 50% v/v supernatants derived from *Pa-Mab* co-cultures as well.

### 2.4 *lasR* complementation

To achieve complementation, low-copy-number plasmids, pJN105 (Newman and Fuqua, 1999) and pJN105.*lasR* (pJN105.L) (Lee et al., 2006) were transformed into chemically competent *lasR* mutant cells as described (Wilder et al., 2009). Plasmid pJN105 served as an empty vector control whereas pJN105.*lasR* expressed *lasR* from an arabinose -inducible ara-BAD promoter. The spent supernatant from *Pa* strains (WT/pJN105, Δ*lasR/*pJN105, and complement Δ*lasR*/*lasR^+^*) was harvested as described earlier except the cultures were induced with 50mM arabinose during growth. *Mab* growth was evaluated in spent supernatants using a luminescence-based 96-well reporter assay (Gupta et al., 2017). Briefly, *Mab-lux* was grown in 50% v/v spent supernatant in a total well volume of 100 µl at a final OD_600_ of 0.01. The plates were incubated at 37°C and the luminescence signal was measured after every 24 h for 2 days. An aliquot was taken for serial dilution and plating to determine CFU for the indicated times.

### 2.5 Heat, protease sensitivity and size fractionation determination of SPAM

To gain insights into the heat stability, size and chemical nature of *Pa*-derived anti-mycobacterial factor known as SPAM, *Mab* growth was examined in heat-denatured, protease treated and size fractionated *Pa* supernatants. For heat treatment, filter-sterilized supernatants were heated at 95°C for 15 min. in a water bath. For protease treatment, cell-free *Pa* supernatant was incubated with 200 µg/ml proteinase K for 2 h at 37°C. Size fractionation of the spent supernatant was carried out by centrifuging through 3kDa MWCO filters (Millipore, SIGMA, UFC900308) for 20 min at 4000 r.p.m. Both retentate and flow-through fractionated samples were filter-sterilized using cellulose acetate, low protein binding syringe filters. *Mab* growth assessment in the heat-denatured supernatant was carried out in flasks as described in the supernatant assay while a high throughput microtiter plate assay described before in *lasR* complementation was adopted for proteinase K (Prot K) treated, and 3kDa size fractioned *Pa* supernatants. Controls including no supernatant and unfractionated/untreated supernatant were included in the assay. The data is an average from 3 biological experiments each with a total of 9 technical replicates.

### 2.6 Total RNA extraction

Total RNA was isolated from mono and co-cultures involving *Pa* and *Mab* after 24 h of incubation at 37°C by using previously reported mycobacterial trizol-based RNA isolation technique (Rohde et al., 2012) with modifications. *Mab* and *P*a cultures were mixed with RNA Protect reagent (Qiagen), mixed by inversion, and centrifuged (4300 r.p.m.) for 20 min. The supernatant was discarded, and the pellet was resuspended in 1 ml RNA Protect reagent before storage at -80°C. The pellets were disrupted using a Mini-Bead Beater 16 (Biospec) 2x at max speed for (for 1 min followed by incubation on ice for 1 min. After this, steps were carried out at 4°C centrifuge as described previously using RNeasy kit (Qiagen) (Rohde et al., 2012). Integrity of isolated RNA was determined using Agilent RNA ScreenTape system.

### 2.7 Dual RNAseq

Transcriptome analysis was carried out with three biological replicates of each sample (mono and co-cultures). Depletion of Ribosomal RNA, mRNA library preparation and sequencing were performed by SeqCenter (earlier MiGS). Library preparation was performed using Illumina’s Stranded Total RNA Prep Ligation with Ribo-Zero Plus kit and 10 bp IDT for Illumina indices. Around Total RNA (275ng) was ribodepleted using oligonucleotide sequences specific for *Pa* and *Mab*. These custom probes are listed in **Supplementary Table 1**. The samples were DNAase treated with Invitrogen DNAase (RNAase-free) and sequencing was performed (NextSeq2000, 2x50bp reads). Sequencing quality control and adapter trimming was performed with bcl-convert (v3.9.3) (https://support-docs.illumina.com/SW/BCL_Convert/Content/SW/FrontPages/BCL_Convert.htm). Read mapping was performed with HISAT2 (Kim et al., 2019) and read quantification was done using Subread’s featureCounts functionality (Liao et al., 2014). Reads for *Pseudomonas* monoculture samples (P1, P2, P3, L1, L2, L3) were mapped to the *P. aeruginosa* PAO1 RefSeq genome NC_002516.2, *Mab* solo culture samples (MB1, MB2, and MB3) to the RefSeq genome GCF_000069185.1 (ASM6918v1), and co-culture samples (PMB1, PMB2, PMB3, LMB1, LMB2, and LMB3) were simultaneously mapped. Differential expression analysis was performed using edgeR’s Quasi-Linear F-Test (qlfTest) functionality against treatment groups. Differentially expressed genes (DEGs) were identified with a cut-off of log_2_ fold-change (FC) (-1 ≥1 to ≤1) and p-value < 0.05 (**Supplementary Table S2**). The KEGG pathway analysis was performed using limma’s (Ritchie et al., 2015) “kegga” functionality with genes with FDR<0.05 (adjusted p-value).

## 3 Results and discussion

### 3.1 *Mab* antagonism in a planktonic co-culture with *Pa*

**Figure 1:**
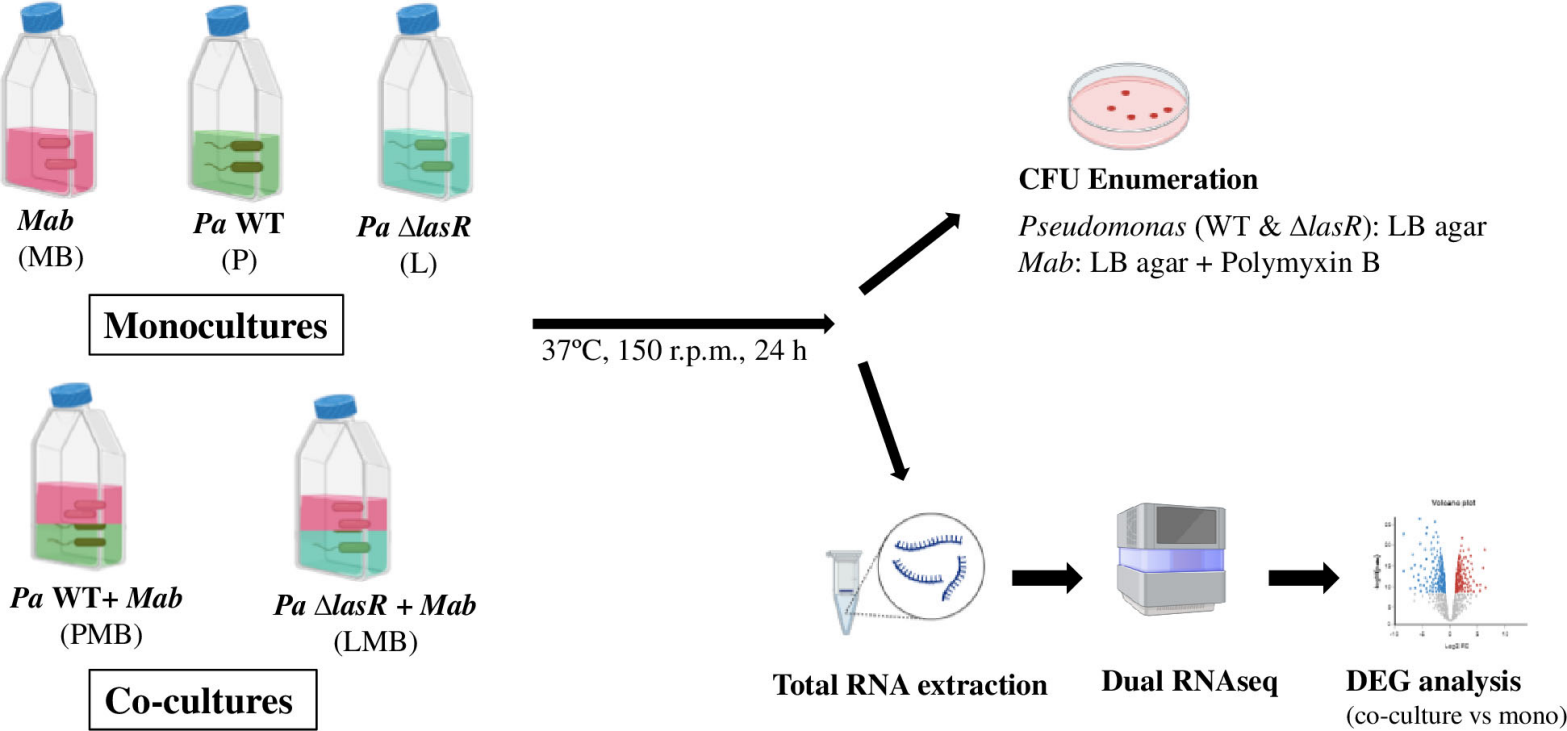
Planktonic co-culture model of *Mab-Pa* interactions. Monocultures of *Mab* (MB) and *Pa* strains (P, L) and co-cultures of *Mab + Pa* WT (PMB) and *Mab* + *Pa* Δ*lasR* (LMB) were grown for 24 h. *Pa* and *Mab* growth was evaluated by plating onto indicated selective media. The mono and co-culture samples were also processed for dual RNAseq analysis.

**Figure 2:**
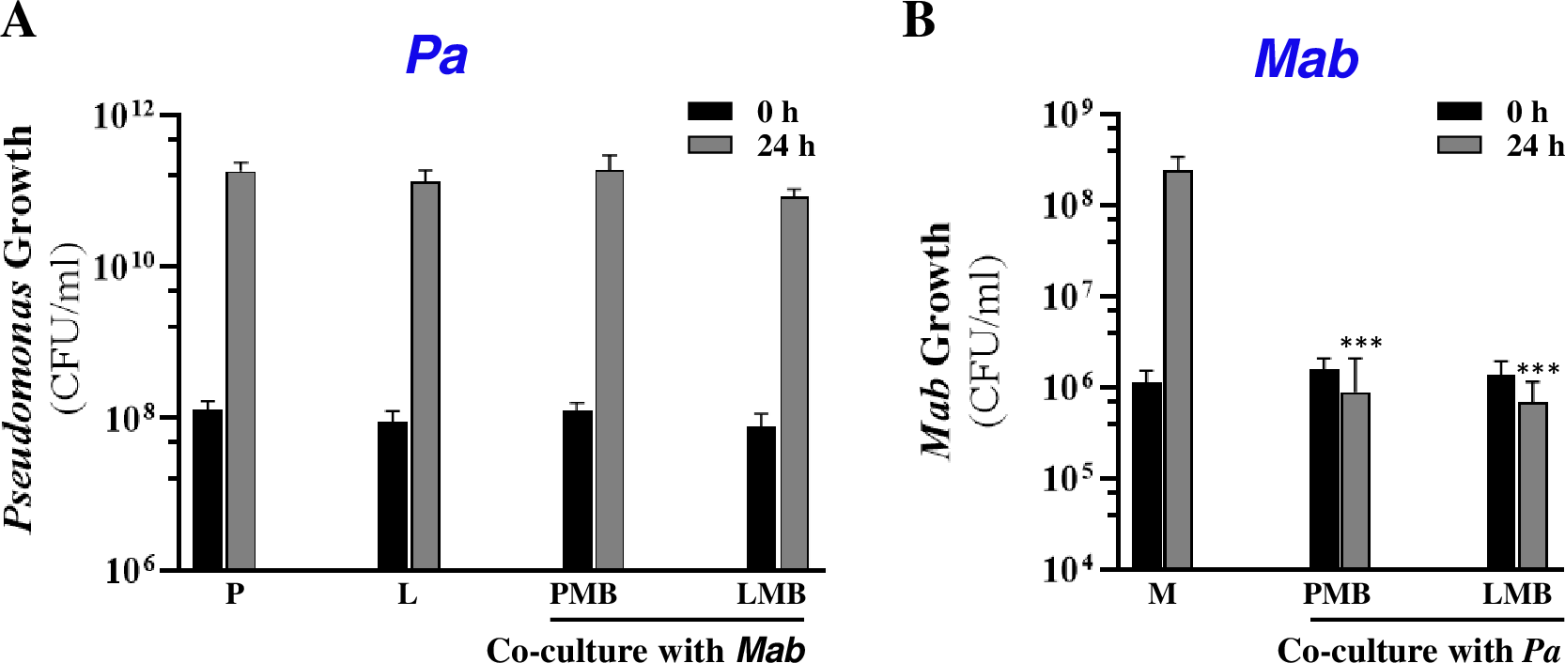
*Pa* antagonizes *Mab*. Growth (CFU/ml) of **(A)** *Pa* and **(B)** *Mab* strains when grown solo and in co-cultures at indicated times. P: *Pa* WT; L: *Pa* Δ*lasR*; MB: *Mab*; PMB: *Pa* WT+ *Mab;* LMB: *Pa* Δ*lasR* + *Mab.* *** denotes statistically significant difference (P<0.0001) (paired t-test) from *Mab* monoculture after 24 h.

To gain an initial understanding of the direct interactions between *Pa* and *Mab*, we compared growth of individual species when present alone (monoculture) and in the presence of the other species (co-culture). A Δ*lasR* strain was also included to assess the role of LasR, a master transcriptional regulator of *Pa* QS in shaping interactions with *Mab*. As noted above, natural *lasR* mutants are frequently isolated from chronically infected CF patients, suggesting dysregulation of QS-regulated virulence factors may offer growth advantage to competitors (Hoffman et al., 2009; Feltner et al., 2016). If LasR-dependent factors are key to *Pa-Mab* interactions, this *in vivo* evolution of *Pa* could significantly impact the progression of *Mab* infections. Using the experimental setup illustrated in **Figure 1**, we evaluated the growth of non-mucoid *Pa* and *Mab* (smooth morphotype) in mono- and co-cultures by CFU enumeration. Co-cultures were started with *Pa* at a lower density (OD600 = 0.05) compared to *Mab* (OD600 = 0.15) to compensate for the faster replication rate of *Pa*. Enumeration of CFU from initial cultures, however, indicated higher CFU/ml of *Pa* which presents a variable that could affect *Pa-Mab* interactions. Growth of both WT and *ΔlasR Pa* strains remained unaffected in the presence of *Mab*. However, in contrast to the 2–3 log increase in CFU observed when *Mab* was grown alone, both WT and *ΔlasR Pa* exerted bacteriostatic antagonism of *Mab* (**Figures 2A, B**). There was no change in the smooth colony phenotype of *Mab* after the co-culturing. The observed antagonism could potentially be attributed to nutrient deprivation of *Mab* in the presence of fast-growing *Pa.* To address this, *Mab* was cultured in 50% water-diluted media to simulate nutrient-limited conditions. After 24 h, *Mab* showed no significant growth differences between the diluted and the undiluted media (**Supplementary Figure 1**), indicating that nutrient limitation did not hamper *Mab* growth. These findings suggest nutrient starvation is unlikely the sole cause of observed *Mab* antagonism in a co-culture with *Pa*. Instead, the primary mechanism underlying bacteriostatic inhibition of *Mab* appears to be a *Pa* derived, LasR-independent mechanism. This observation is in contrast to a recent report where no *Mab* antagonism was observed in the planktonic co-culture with *Pa*, even when different *Pa: Mab* ratios were tested (Idosa et al., 2022). This discrepancy may be attributable to differences in media, bacterial strains or culture conditions. The observed *Mab* growth arrest in the presence of *Pa* in this study indicates that *Mab* may adopt a non-replicating persister-like state as a strategy to counter *Pa* antagonism.

### 3.2 Secreted Pa factor(s) are bactericidal toward Mab

**Figure 3:**
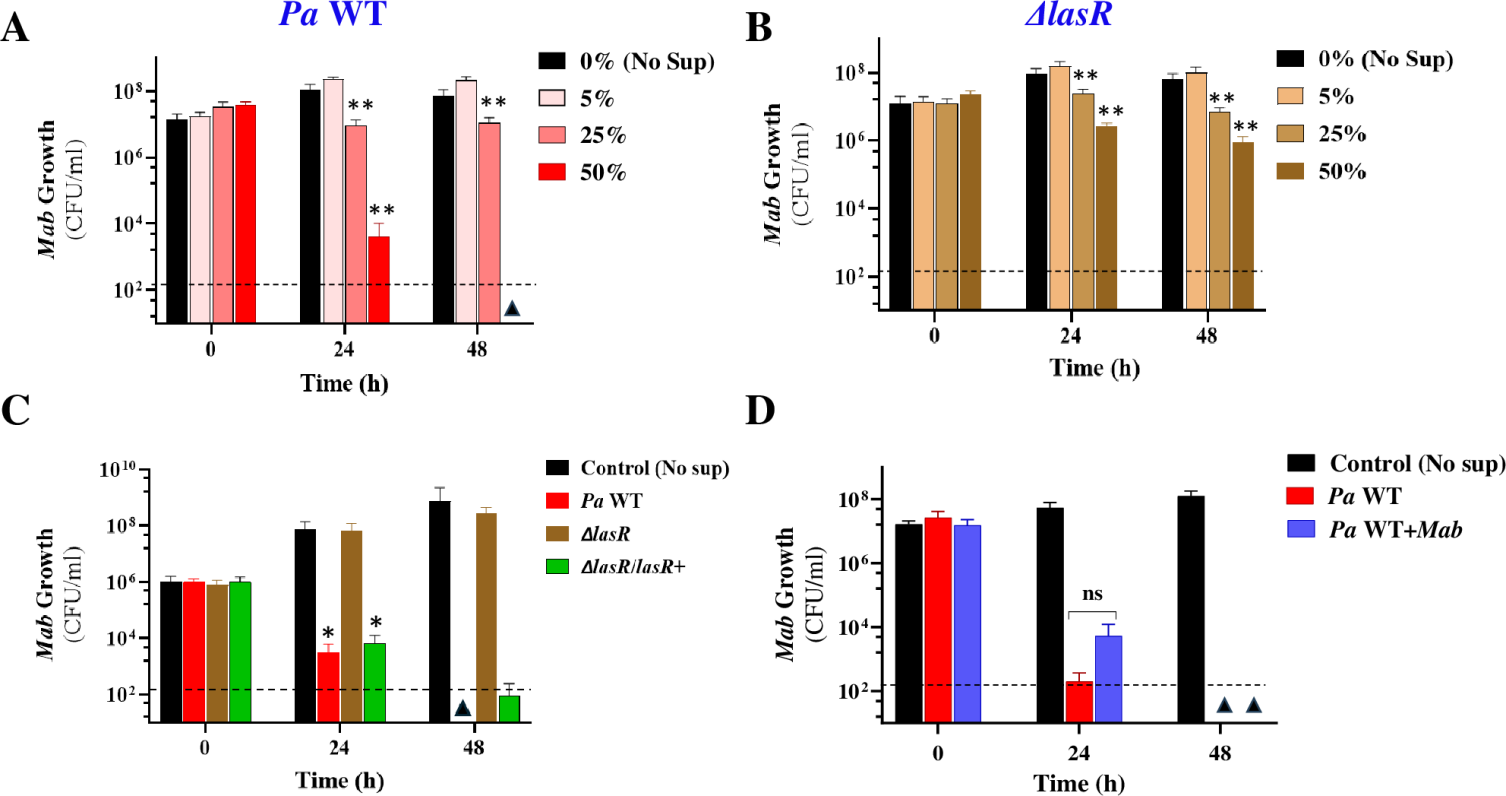
*Mab* viability in spent *Pa* supernatants. *Mab* growth (CFU/ml) in media supplemented with **(A)** *Pa* WT and **(B)** *Pa* Δ*lasR* culture supernatants at 0%, 5%, 25% and 50% concentration (v/v) **(C)** *Mab-lux* growth in 50%v/v spent *Pa* supernatants after complementation of *lasR* mutation. A *lasR* allele was expressed from an arabinose inducible promoter carrying plasmid, pJN105. *Pa* WT and *Pa* Δ *lasR* strains carry the empty plasmid **(D)** *Mab* growth in sup obtained from *Pa* WT mono and co-cultures with *Mab*. * P<0.01, ** P<0.001 from control, ns, insignificant (paired t-test). The dotted line denotes the limit of detection (LoD) (2*10^2^ colonies) and triangles denote samples where CFU was below LoD.

Next, we investigated whether *Mab* inhibition by *Pa* in co-cultures was mediated by a contact-dependent mechanism, as previously reported, or secreted factors. To address this, we examined the effect of *Pa* cell-free culture supernatant on *Mab* growth. When *Mab* was exposed to a range of concentrations (0%, 5%, 25% and 50% v/v) of *Pa* WT supernatant (sup), a dose- and time-dependent reduction in CFU was observed (**Figure 3A**). Exposure to 50% (v/v) *Pa* WT sup resulted in a 3–4 log decline in CFU compared to the control (no sup) within 24 h and complete sterilization within 48h, a bactericidal effect not even observed with first-line antibiotics used for clinical treatment of *Mab* infections (Maurer et al., 2014). This indicates the presence of a highly potent mycobactericidal compound. However, this drastic cidal phenotype was lacking with the *Pa* Δ*lasR* supernatant, which caused only minor ~1-log reduction in CFU at 48h (**Figure 3B**). Heterologous expression of the *lasR* allele from an arabinose inducible promoter in the *Pa* Δ*lasR* strain restored the cidal activity as indicated by the decline in *Mab* CFU and luminescence signal (**Figure 3C**; **Supplementary Figure 2**). This suggests that a LasR-dependent secreted factor (directly or indirectly regulated) mediated the potent cidal activity against *Mab.* We designated this cidal factor as SPAM (**S**ecreted ***P*** *seudomonas* **A**ntagonist of ***M*** *ab*) throughout the rest of this study.

The striking susceptibility of “naïve” *Mab* to killing by secreted *Pa* factors versus the tolerance of *Mab* when grown in co-culture with *Pa* suggested that interspecies interactions have either downregulated the production of cidal factors by *Pa* or rendered *Mab* more resistant to their effects, or both. To investigate these hypotheses, we explored the possibility of 1) downregulation of *Pa* secreted virulence factors and 2) *Mab* adaptation during co-culturing by evaluating *Mab* growth in 50% v/v spent supernatant from co-cultures as well. The fact that supernatants from *Pa* co-cultured with *Mab* exerted a cidal activity against *Mab* comparable to that observed with supernatant from *Pa* monoculture supports the conclusion that *Mab*-induced downregulation of SPAM did not occur in the co-culture (**Figure 3D**). The remaining likely explanation is that *Mab* mounts adaptative responses to counter killing by *Pa* during their side-by-side growth. The molecular adaptation of *Mab* in response to *Pa* presence was explored through dual RNAseq analysis later in the study.

### 3.3 SPAM characterization

**Figure 4:**
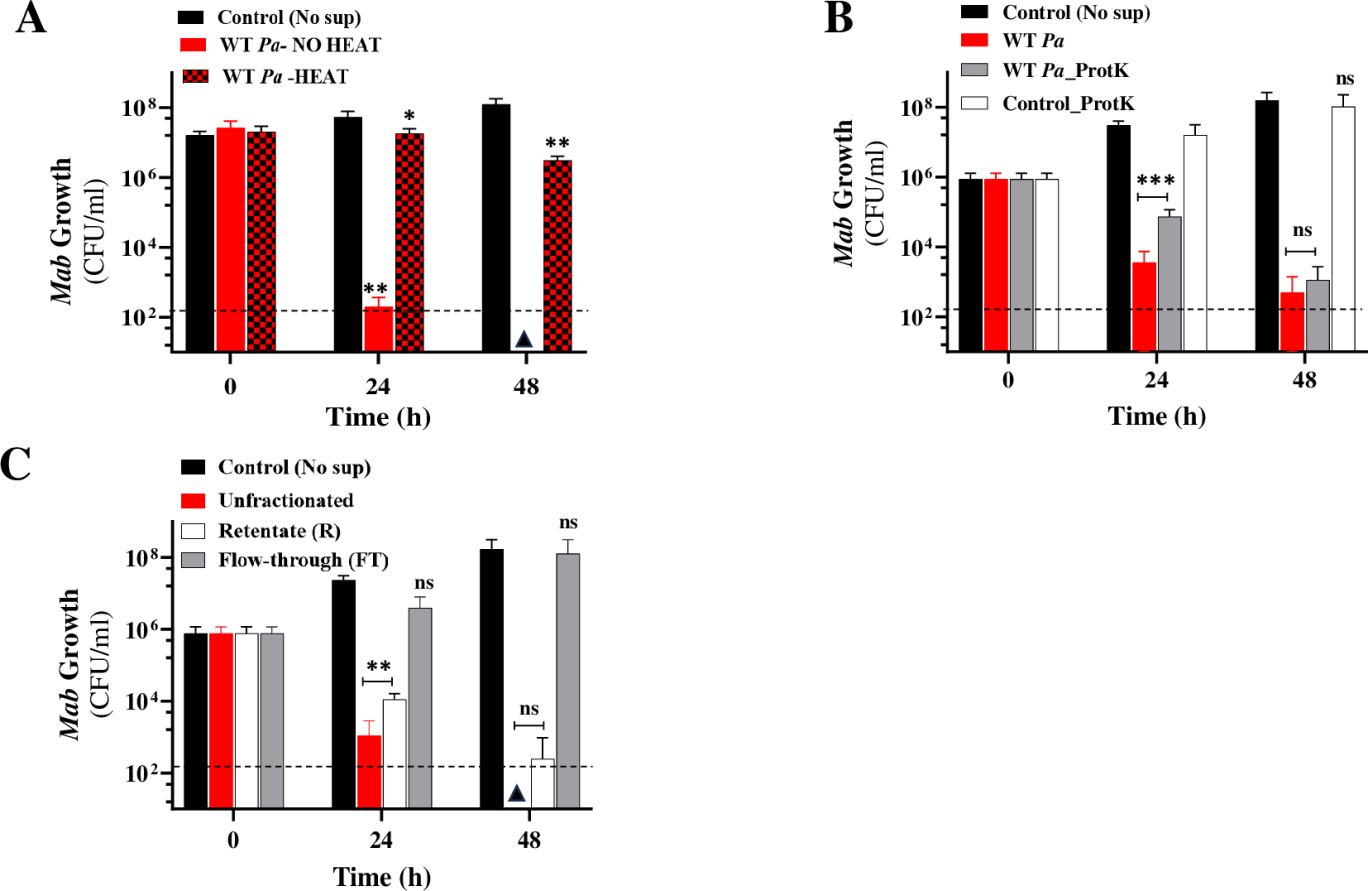
SPAM characterization. *Mab* growth (CFU/ml) in WT *Pa* supernatant (50% v/v) **(A)** with and without heat denaturation **(B)** proteinase K (ProtK) treated **(C)** Size fractionated (3kDa MWCO). The data is an average from at least three experiments. Note: For panel B and C, 10^1^ is the lowest dilution plated. * P<0.01, **p <0.001 from control, ***P=0.0001, ns, non-significant (two-tailed paired t-test). The dotted line denotes the limit of detection (LoD) (2*10^2^ colonies) and triangles denote samples where CFU was below LoD.

To characterize the potent SPAM factor, we assessed *Mab* growth in heat-denatured, protease-treated, and size-fractionated spent *Pa* supernatant. Heat inactivation of *Pa* (WT) supernatant largely abolished the cidal activity indicating the heat-labile nature of SPAM (**Figure 4A**). To determine whether SPAM is proteinaceous in nature, *Pa* sup was pretreated with proteinase K (Prot K) and viability of a *Mab-lux* reporter strain was evaluated through two readouts - luminescence and CFU. Prot K is a broad-spectrum serine protease that cleaves peptide bonds next to the carboxyl-terminal of aromatic and hydrophobic residues (Bajorath et al., 1988). Two controls, untreated and Prot K treated media were included. The protease treatment did not eliminate the cidal activity of *Pa* supernatant (**Figure 4B**; **Supplementary Figure 3A**). Both the untreated and Prot K digested cultures showed a ~4–5 log decrease in *Mab* CFU after 48 h compared to controls (**Figure 4B**). These results strongly suggest that SPAM is not proteinaceous in nature, although we cannot definitively rule out a protein factor involvement because some *Pa* secreted proteins may resist degradation by Prot K digestion (Bajorath et al., 1988). Size fractionation of supernatant indicated that the potent cidal SPAM factor is >3kDa in size as the retentate fraction killed *Mab* but not the flow-through fraction (<3kDa) (**Figure 4C**; **Supplementary Figure 3B**). This characterization would eliminate small molecules like phenazines or respiratory toxins (e.g. HQNO) as prime candidates, unless they were encapsulated in outer membrane vesicles as reported previously (Vrla et al., 2020). Further investigation will be required to isolate and identify this LasR-controlled interspecies antagonist.

Overall, our co-culturing and supernatant assays investigated direct and indirect *Mab-Pa* interactions which demonstrated a *Pa*-derived negative impact on *Mab* growth. Static growth of *Mab* when in physical contact with *Pa* appears to be mediated by either a *las* independent or a non-QS mechanism whereas cidal activity during indirect interaction with *Pa* is mediated via Las QS dependent secreted factors.

### 3.4 *Mab* adaptation at a molecular level during co-culturing with *Pa*

**Figure 5:**
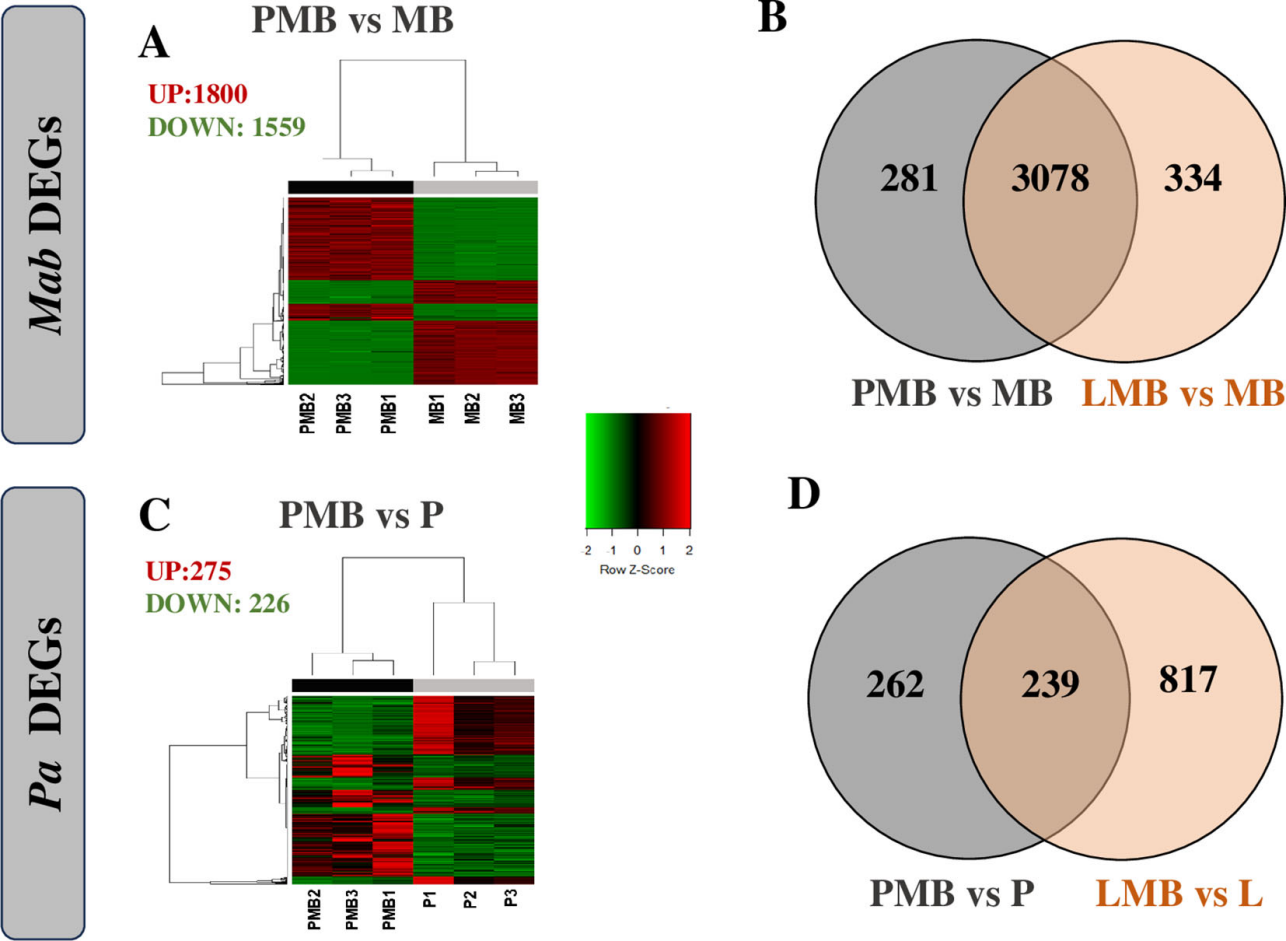
Transcriptomic responses of *Mab* **(A, B)** and *Pa* **(C, D)**. Differentially expressed genes of *Mab* **(A)** and *Pa* **(C)** normalized counts per million of three replicates are represented by heat map. Venn diagrams represents overlap in *Mab* expression when cultured with WT and *lasR Pa* **(C)** and in *Pa* expression when cultured with *Mab* **(D)**. P: *Pa* WT, L: Δ*lasR*, MB: *Mab*, PMB: WT *Pa*-*Mab* co-culture, LMB: Δ *lasR* -*Mab* co-culture.

To explore molecular cross talk between the two pathogens during co-culturing, we carried out dual RNAseq analysis and compared the changes in gene expression in both *Pa* and *Mab* in a co-culture vs monoculture after 24h, corresponding to the planktonic co-culture assay point (**Figure 1**). We achieved a sequencing depth of 20–80 million total reads per sample meeting the minimum (10^6^) reads requirement for a comprehensive differentially expressed genes (DEGs) analysis (Toung et al., 2011; Westermann et al., 2016). The differentially regulated *Mab* and *Pa* genes are listed in **Supplementary Table 2** and depicted with heat maps and Venn diagrams (**Figure 5**; **Supplementary Figure 4**). Notably, the *Mab* transcriptome exhibited much more dramatic changes than *Pa* during co-culture of these two CF pathogens. More than 68% of the coding genes of *Mab* (3359/4920) were differentially regulated in the presence of WT *Pa*. Out of a total of 3359 *Mab* DEGs, 1800 were up-regulated and 1559 were down-regulated whereas a mere 275 and 226 Pa genes showed up and down-regulation, respectively (**Figures 5A, C**).

The transcriptional profile of *Mab* and *Pa* genes did not show any striking differences when the Δ*lasR Pa* strain was employed. *Mab* showed altered regulation of 3412 genes with up-regulation of 1866 and down-regulation of 1546 when co-cultured with Δ*lasR* (**Supplementary Figure 4A**). Like WT *Pa*, Δ*lasR* showed relatively modest changes with 488 and 568 genes up- or down- regulated, respectively (**Supplementary Figure 4B**). The Δ*lasR* strain showed downregulation of signature genes including direct LasR-dependent genes (*lasI, lasR, lasA, apr, rsaL*), phz2 operon, *hcnAB, qscR, rsaL*, as well as LasR controlled *rhl*-system (*rhlR, rhlAB*), and *pqs* system genes (*pqsH*) as expected with loss of this QS signal receptor (**Supplementary Table 2**).

Overall, the extensive transcriptional reprogramming of *Mab* during co-culture with *Pa* reflects adaptive responses that likely contributed to *Mab’s* ability to withstand potentially cidal *Pa* secreted factors, whereas *Pa* appears to remain relatively undeterred during this rather one-sided interspecies interaction.

### 3.5 Co-culture with *Pa* triggers slow-growing persister-like state in *Mab*

**Figure 6:**
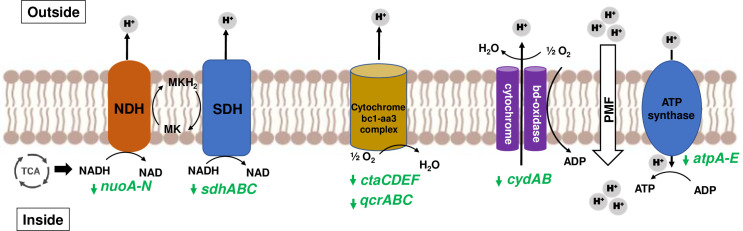
Slowing of *Mab* respiration during co-culturing. Mycobacterial NADH from TCA cycle enters mycobacterial electron transport chain (ETC) and supplies electrons to NADH dehydrogenase (NDH) and succinate dehydrogenase (SDH) to reduce menaquinone (MK) to menaquinol (MKH_2_). Cytochrome oxidases (bc1-aa3 complex and bd-type) oxidize back MK by transferring electrons to oxygen. Proton motive force (PMF) generated due to proton translocation is utilized by ATP synthase to produce ATP. Genes encoding ETC components are represented in green, and downward arrow denotes downregulation. FC values of affected genes are listed in **Supplementary Table S3** under Electron Transport Chain heading.

Persistence, which is characterized by slow or nonreplicating state, leads to antibiotic tolerance requiring prolonged treatment and high chances of treatment failure and recurring infections. Slow growth is marked by down-regulation of DNA replication, transcription and translation machinery, cell-division processes, electron transport, ATP-proton motive force and other cell processes to conserve energy (Beste et al., 2009). Differential gene expression analysis between *Mab-Pa* co-cultures vs *Mab* monoculture revealed decreased carbon flux into metabolic pathways, reduced respiration, and energy metabolism (**Supplementary Table 3**, **Supplementary Figure 5**). Genes involved in cell division (*ftsZ*, *fts*K, *fts*X, *fts*E, *fts*H, and *ftsW*), DNA replication (*dnaA*, *dnaN*, *gyrA, gyrB*), and transcription and translation (*fusA*, *tuf*, *tsf*, *rpoA, rpoB)* were repressed in the presence of *Pa.* A non-functional LasR did not change this response as *Mab* co-cultured with the Δ*lasR* exhibited a similar transcriptomic profile. There was also down-regulation of type-I NADH dehydrogenase encoded by *nuoA-N* genes, succinate dehydrogenase (*sdhABC*), cytochrome oxidases, and F_0_-F_1_ ATP synthase genes (*atpA-E*) (**Figure 6**; **Supplementary Table 3**). This transcriptional response in *Mab* is akin to changes observed in *M. tuberculosis* in its non-replicating persister phase (Shi et al., 2005; Maurya et al., 2018). Entry of *Mab* into a non-replicating state could be a way to endure hostile conditions generated by *Pa* under co-culture conditions. This slow-growing state could have clinical implications, by contributing to the disconnect between *in vitro* and *in vivo* antibiotic susceptibility and to the ineffectiveness of current treatment regimens.

### 3.6 High induction of HGT elements in *Mab* during co-culture

**Figure 7:**
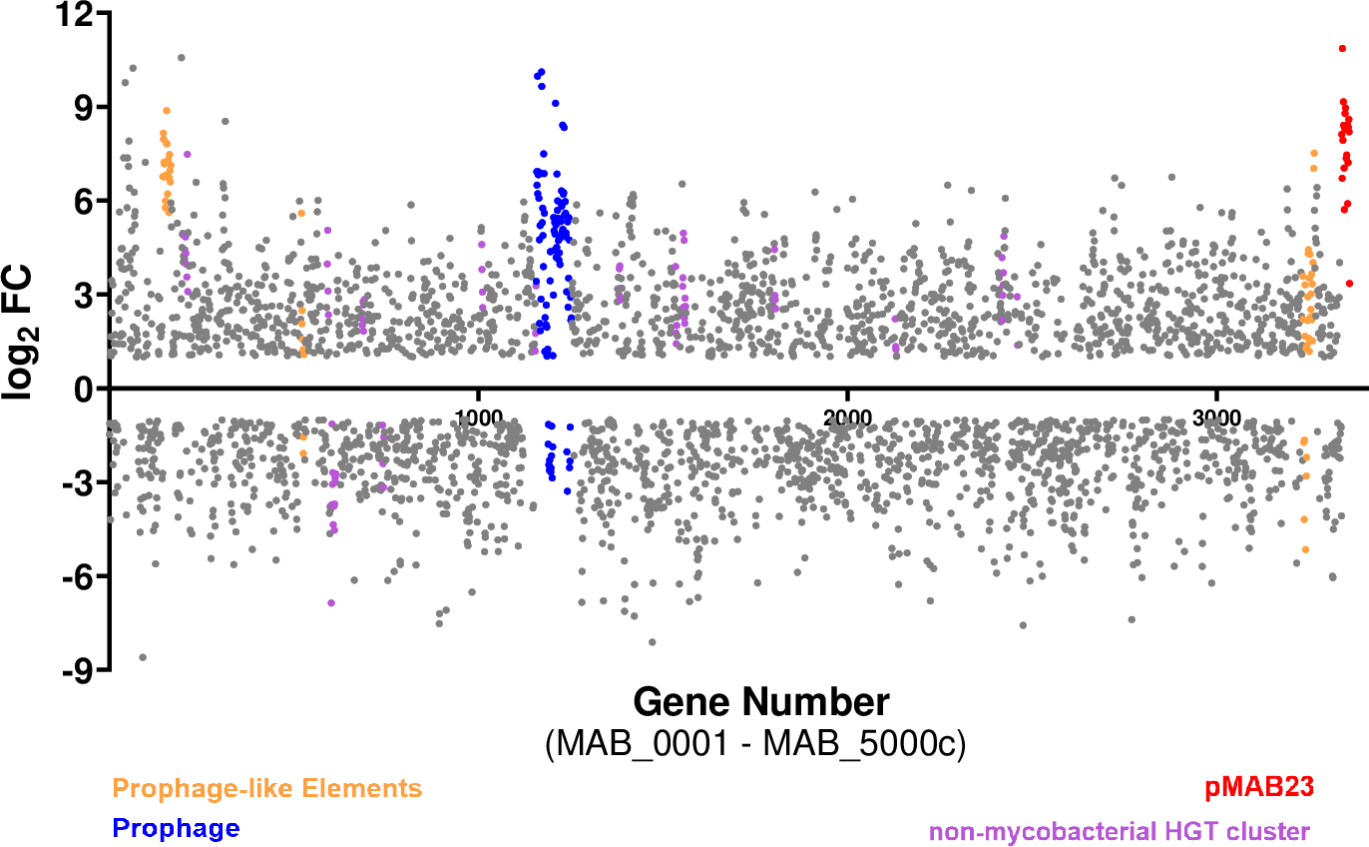
Up regulation of *Mab* HGT elements in a co-culture. A scatter plot showing differentially regulated genes in *Mab* (PMB vs MB) where each dot represents a differentially expressed gene. The colored dots are the putative HGT acquired genes.

The *Mab* genome contains a 23-Kb plasmid, pMAB23, and several other gene clusters predicted to be acquired by horizontal gene transfer (HGT) from closely related mycobacterial species and from non-mycobacterial community (Ripoll et al., 2009). Strikingly, mobile elements, prophage and prophage-like elements and other HGT acquired genes including those putatively derived from *Pseudomonas* sp. were highly upregulated, with some having a log_2_ FC as high as 10. All 22 genes (MAB_p01 to MAB_p22c) encoded on the pMAB23 plasmid including the essential gene, *repA* (MAB_p16c), and putative arsenate resistance conferring genes were induced (**Figure 7**; **Supplementary Table 4**). In addition, several *Mab* genes in a 61 kb prophage region (Dedrick et al., 2021) (MAB_1723 to MAB_1833) exhibited strong transcriptional response in the presence of *Pa* (both WT and the *lasR* mutant). Three-prophage like elements (Ripoll et al., 2009) and 17-gene clusters acquired from the non-mycobacterial community were also differentially regulated. Out of the 17-clusters, 13 showed upregulation and only two, involved in degradation of phenylacetic acid (MAB_0899c-0911) and DNA (MAB_1093c-1098), respectively were down-regulated (**Figure 7**; **Supplementary Table 4**). The increased expression of mobile elements, prophage and other HGT genes in *Mab* under co*-*culture conditions suggest functional importance beyond being just passive elements in the genome. Increased expression of prophage genes is associated with survival under certain environmental conditions such as low pH in *M. avium* (Miao Zhao et al., 2016). A transcriptomic response including higher expression of transposable elements, plasmid and viral-borne genes has been reported in other organisms such as slow-growing yeast cells for driving diversity for future stressful conditions (van Dijk et al., 2015).

### 3.7 Remodeling of central carbon metabolism of *Mab* in presence of *Pa*

**Figure 8:**
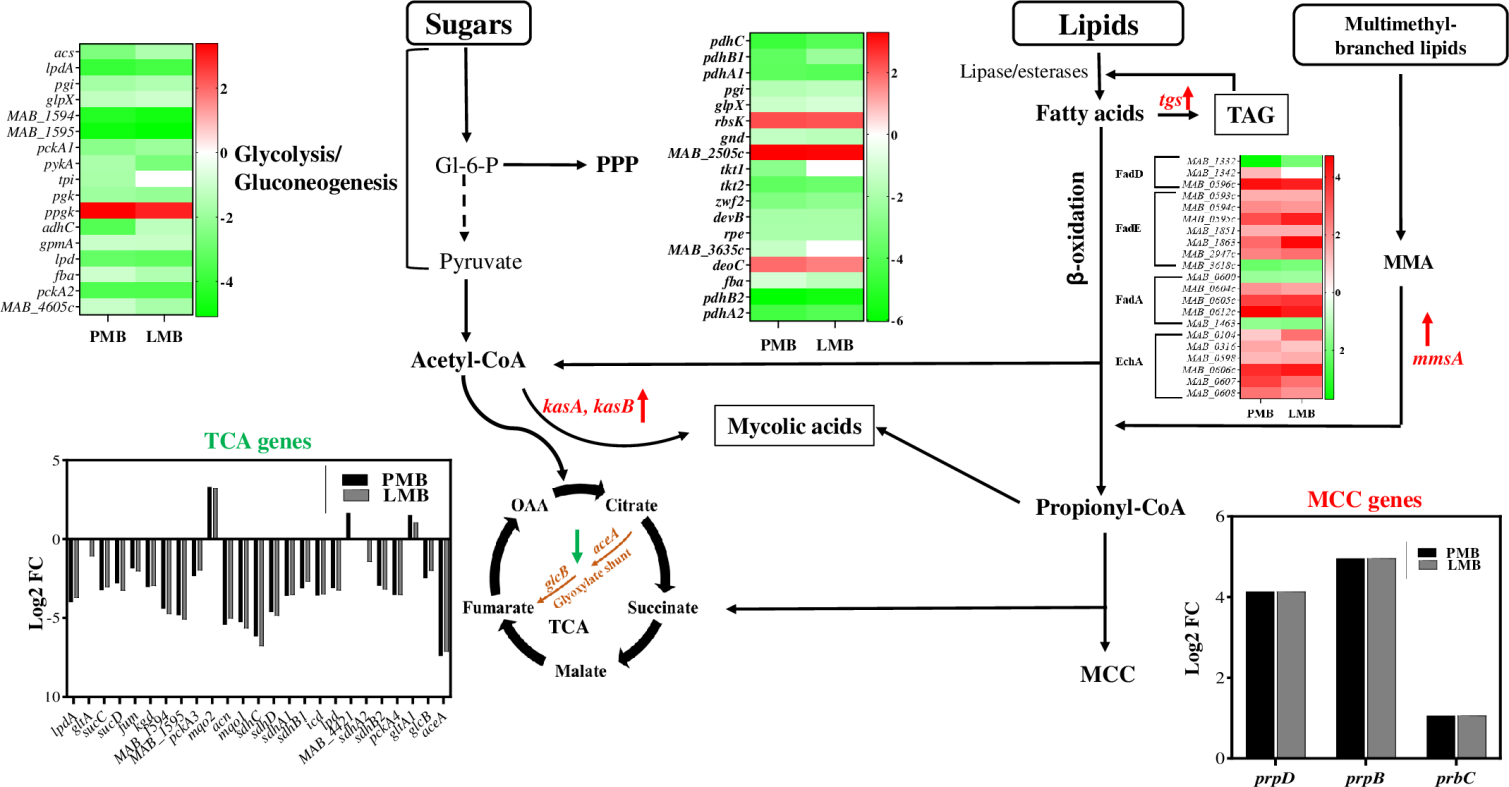
Carbon flow switch from sugars to fatty acids during co-culturing. The figure depicts an integrated view of central carbon metabolic pathways with differential expression of associated genes *via* heat maps, bar graphs, and by colored up and down arrows. PPP, pentose phosphate pathway; TCA, tricarboxylic acid cycle; OAA, oxaloacetate; MCC, methyl citrate cycle; TAG, triacylglycerol; MMSA, Methylmalonate semialdehyde. Green and red arrow denotes down and upregulation, respectively. Carbon flow shunt to mycolic acids and TAG storage (boxed).

One of the major transcriptional responses noted in *Mab* when co-cultured with *Pa* was in central metabolic pathways. RNAseq data reflected a switch in carbon flow from sugars to fatty acids during co-culturing, with downregulation of glycolysis, gluconeogenesis, and Pentose Phosphate Pathway (PPP) pathways. The key enzymes involved in these pathways such as NADP-dependent dehydrogenase, aldolase, isomerases, and ATP-generating phosphoglycerate kinase, fructose 1,6-bisphoatase (*glpX*) and PEP carboxykinase (*pckA*) (**Figure 8**; **Supplementary Table 5**) were downregulated. This indicates reduced energy and biosynthetic precursors demand, aligning with the *Mab* phenotypic shift from replicating to a slow growing phase. Along these same lines, energy-consuming anabolic pathways were downregulated while lipid catabolism increased. There was an upregulation of lipases and fatty acid β-oxidation genes, along with decreased fatty acid synthesis (**Supplementary Table 5**), which suggest lipid breakdown for membrane biosynthesis or storage as triacylglycerol (TAG) for persistence and future energy sources. The elevated transcripts of methylcitrate cycle (MCC) genes (*prpDCB*) and methylmalonate-semialdehyde dehydrogenase encoded by *mmsa* (log_2_ FC: 4) indicate propionyl CoA accumulation and subsequent detoxification (Munoz-Elias et al., 2006; Savvi et al., 2008) (**Figure 8**; **Supplementary Table 5**). Reduced expression of propionyl-CoA carboxylase (*accD5*), methylmalonyl-CoA mutase (*mutB*) genes reflect preferential utilization of MCC over methyl malonyl (MM) for propionyl detoxification (**Supplementary Table 5**). Upregulation of mycolic acid synthesis genes (MAB_2028-MAB_2031 and beta-keto acyl synthases) hints at the incorporation of acetyl and propionyl CoA into mycolic acids. Key enzymes, *kasA* and *kasB* with a role in elongation of meromycolate chain showed upregulation (**Supplementary Table 5**). In addition, upregulation of triacylglycerol synthases (*tgs*) indicates TAG synthesis. Three out of 7 *tgs* genes encoded in the *Mab* genome (MAB_0854, MAB_1278, and MAB_2964), exhibited increased expression. This differential induction of *tgs* is also noted in *M. tuberculosis* under different inducing environments. Out of 15 *tgs* encoded in *Mtb*, *tgs1* was upregulated under hypoxia/nitric oxide stress condition whereas *tgs2* was induced by low pH (Viljoen et al., 2016; Maurya et al., 2018). TAG accumulation is a hallmark of persisting *Mtb* which aids in drug tolerance and survival in the host (Maurya et al., 2018).

Based on transcriptomic data, we speculate that excess fatty acids are channeled into intracellular lipids accumulation in the form of triacylglycerols (TAG) which can be hydrolyzed back by lipases when needed. TAG synthesis serves as a storage sink for persistence and survival under stress such as co-culture conditions. Resource limitation and competition during co-culture, perhaps enhanced by the lack of ample available sugars and other catabolizable carbon sources in LB medium (Sezonov et al., 2007), may be one driver of fatty acid catabolism and rerouting of carbon flow towards storage and alterations in cell wall composition to maintain cell viability and integrity. Similar carbon flow re-routing has been noted during *Mtb* infection (Garton et al., 2008; Russell et al., 2009) and has implications in persistence and drug tolerance. The phenotypic growth arrest and persister-like metabolic remodeling may also reflect the impact of respiratory toxins and other antimicrobial metabolites secreted by *Pa*. Secreted *Pa* factors like HCN, pyocyanin, and quinolone N-oxides inhibit *Staphylococcus aureus* growth by blocking respiration, leading to a small-colony persister phenotype (Biswas and Gotz, 2021).

### 3.8 Co-culturing of *Mab* and *Pa* triggers competition for Fe

**Figure 9:**
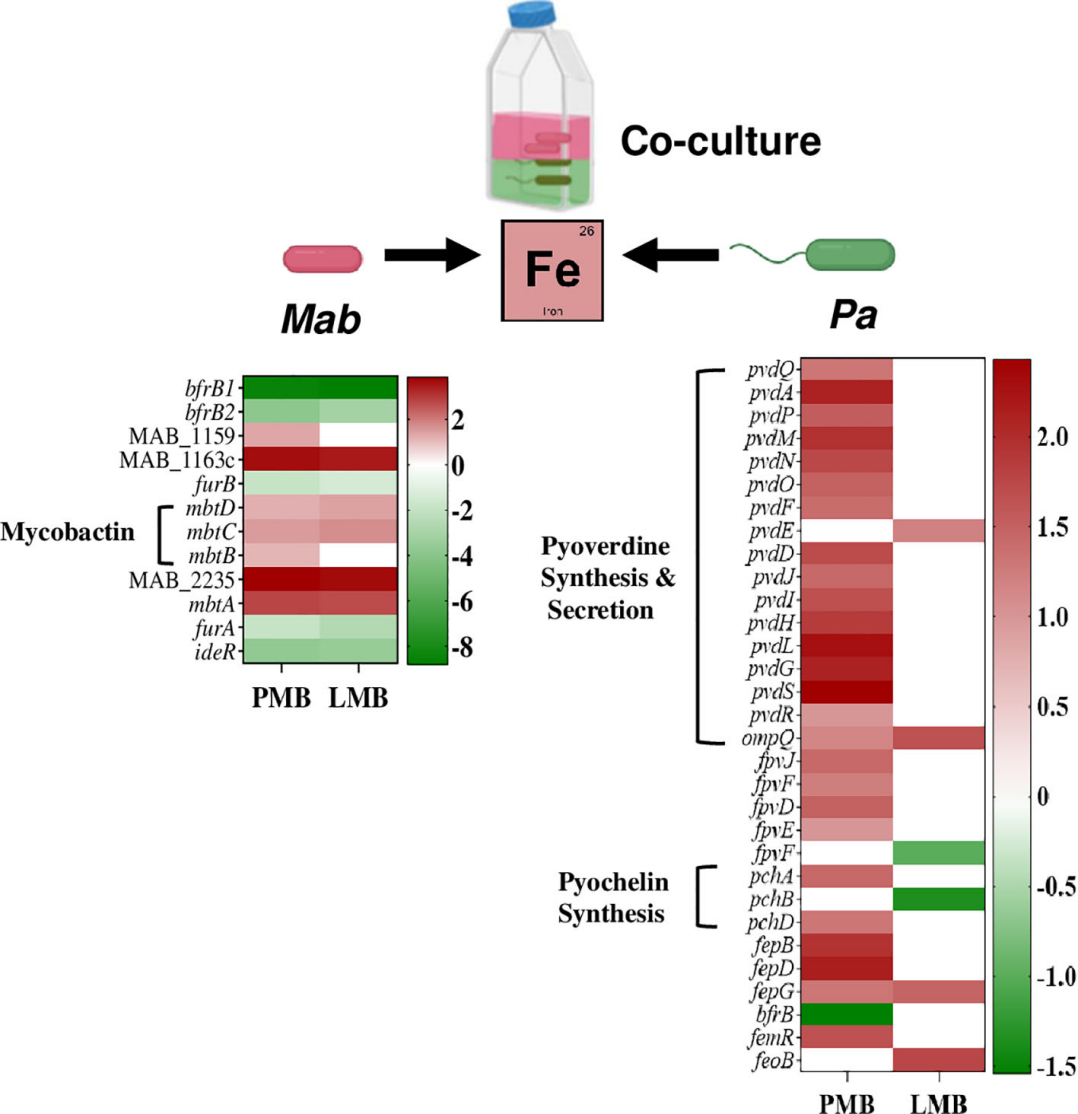
Competition for Fe among *Mab* and *Pa*. The heat map shows up and down regulated iron-responsive genes of *Mab* and *Pa* (WT and Δ*lasR*) during co-culture.

Culturing *Mab* and *Pa* together forces them to compete for common essential nutrients including Fe to replicate and survive. *Pa* utilizes several strategies to acquire iron under infection conditions including production and secretion of siderophores (Lamont et al., 2009; Martin et al., 2011). Transcriptional profiles from dual RNAseq showed upregulation of siderophore-mediated iron-uptake, and downregulation of iron-storage in both bacterial pathogens, indicative of a Fe-starvation response (**Figure 9**; **Supplementary Table 6**). This pattern of altered expression of iron-responsive genes in both *Pa* and *Mab* in our co-culture model is indicative of competition between the interacting pathogens for Fe and likely other essential nutrients.

*Pa* produces two extracellular Fe chelating siderophores called pyoverdine and pyochelin with high and low affinity to Fe, respectively. Pyochelin biosynthesis genes, *pchA* and *pchD* and the gene cluster involved in synthesis and maturation of the major siderophore, pyoverdine (*pvdQAPMNOFEDJIHLG*) showed elevated expression in WT *Pa* when co-cultured with *Mab* (**Figure 9**; **Supplementary Table 6**). Besides synthesis, genes involved in secretion of pyoverdine into the extracellular environment via an ATP-dependent *pvdRT-ompQ* also had elevated expression. The transcript levels of the regulatory gene *pvdS*, encoding an iron-starvation sigma factor known for positive regulation of pyoverdine synthesis, was also enhanced. Downregulation of *bfrB* (bacterioferritin), which is involved in Fe storage, is also consistent with Fe-limiting conditions. Siderophore-mediated Fe uptake and storage genes were not differentially regulated in *Pa* in the Δ*lasR Pa-Mab* coculture (**Figure 9**; **Supplementary Table 6**) as expected because siderophore production is a QS regulated phenomenon (Stintzi et al., 1998; Venturi, 2006; Lin et al., 2018).

Similar to the Fe-limited transcriptional profile seen for *Pa* in co-culture, *Mab* also upregulated genes associated with iron uptake and downregulated iron storage genes. Genes encoding for the synthesis of the mycobacterial siderophore, mycobactin *(mbtDCBA)* showed higher expression whereas iron-dependent regulators (IdeR, FurA, and FurB) and the two putative Fe storage associated bacterioferritin B genes (MAB_0126, and MAB_0127) were downregulated (**Figure 9**; **Supplementary Table 6**).

The iron sequestering response from co-culture partners (*Pa* WT and *Mab*) indicates competition and survival efforts. Understanding Fe competition is vital for studying bacterial interactions, pathogenesis, and evolution of microbial communities. The adaption of various Fe –uptake strategies by both *Mab* and *Pa* in response to the co-culture environment is reminiscent of the human host infection conditions where the scarce availability of iron forces pathogens to adapt to survive and establish a successful infection. Effective consumption of limited nutrients by pathogens in CF lung will influence interaction dynamics and shape disease progression.

### 3.9 Interspecies interaction between *Mab* and *Pa* alters virulence factor expression

Dual transcriptomic profiling revealed that the well-known virulence factors of *Pa* including both surface-associated and secreted factors did not show notable up or downregulation except for pyocyanin and siderophores (**Supplementary Table 7**). Our *in vitro* assays with *Pa* spent supernatant from mono and co-cultures also indicated no significant *Mab*-mediated downregulation of *Pa* secreted virulence factors.

However, in *Mab*, the presence of *Pa* triggered increased expression of several virulence factors such as MmpL/MmpS family lipid transporters, lipoproteins, and drug resistance associated genes involved in efflux and target modification (**Supplementary Table 7**). Several monooxygenases/dioxygenases implicated in ring cleavage of aromatic compounds were upregulated indicating *Mab* potential to inactivate *Pa* aromatic compounds such as pyocyanin and QS signals (**Supplementary Table 8**). Seventeen out of twenty-three dioxygenases present in *Mab* genome were up-regulated including the *aqd* cluster, MAB_0301-MAB_0303, known to degrade *Pa* AQ signals (Birmes et al., 2017; Birmes et al., 2019) (**Supplementary Table 8**). Also, elevated expression of cyanate hydratases encoded by MAB_0054c and MAB_2545c may indicate induction of *Mab* defenses against HCN produced by *Pa* (Letoffe et al., 2022; Nolan and Allsopp, 2022). This tailored response of *Mab* to *Pa* virulence factors indicates *Mab* adaptation efforts to survive to coexist with *Pa* in similar niches within CF lung.

## 4 Conclusion

In this study, we exploited both culture–based and dual RNA transcriptomics strategies to explore interactions between *Pa* and *Mab*, two difficult-to-treat pathogens that commonly afflict CF patients. A handful of prior studies utilizing solid surface biofilm models (Rodriguez-Sevilla et al., 2019; Idosa et al., 2022) have reported *Pa-*mediated antagonism of *Mab*, although the molecular mechanisms underlying this interaction remain unknown. In contrast to the recent findings of Idosa et al (Idosa et al., 2022), this study reports *Pa* antagonism of *Mab* in a planktonic co-culture model, with *Pa* exerting a strong bacteriostatic effect on *Mab*. We also discovered the presence of soluble secreted factor(s) in *Pa* supernatants, which we dubbed SPAM, that exhibited potent bactericidal activity against *Mab.* Initial efforts to characterize SPAM indicate a protease-resistant, heat-labile factor of >3Kda whose expression is dependent on the LasR QS system. Isolation and identification of this natural product antimycobacterial agent, which is beyond the scope of the current study, may yield a valuable starting point for development of new antimicrobials and a chemical biology tool for identification of novel drug targets in *Mab.* Several of our key observations were partially corroborated by Nandanwar et al. (2024) in a study of *Mab*-*Pa* interactions published during the final preparation of this manuscript, after publication of our corresponding pre-print (Gupta et al., 2024). Specifically, the bacteriostatic effect of *Pa* on *Mab* and unperturbed growth of *Pa* were confirmed by similar co-culture experiments. They noted the inconsistent inhibitory effect of filtered *Pa* supernatants towards *Mab*, with either decrease (~1.5log) in CFU over 72h in one figure, or an increase (~1-log) in CFU within 72h in another (vs ~3-log growth of untreated *Mab*). This contrasts with our data showing potent bactericidal effect on *Mab* cultures with no recovery of colonies after 48h. The identification of this *Pa*-derived anti-mycobacterial factor(s) as well as the reason for differences in bactericidal potency between the two studies remain to be elucidated.

The ability of *Mab* to withstand SPAM-mediated killing and persist alongside *Pa* in the co-culture model, despite being outnumbered ~100:1 from the outset, serves as evidence that interspecies interactions induce alternations in *Mab* that facilitate persistence. To gain genome-wide insight into global transcriptional changes triggered by *Pa-Mab* interactions, we conducted what is to our knowledge the first dual RNAseq analysis of these CF pathogens. *Mab* was notably more strongly impacted than *Pa* (based on number of differentially expressed genes), in line with *Pa’s* dominant role as a “bacterial bully” in its interactions with other species. In addition to *Mab* genetic signatures consistent with persistence and growth arrest we observed, there was evidence of adaptative responses to thwart known antimicrobial effectors of *Pa*. This valuable dataset provides novel insights into *Pa-Mab* interspecies interactions, however, much more work is needed to understand how these interspecies dynamics impact clinical outcomes and treatment. The apparent ability of *Mab* to withstand potentially bactericidal effectors secreted by *Pa* is likely critical for *Mab* to co-exist with *Pa* during chronic infections of vulnerable patients with CF or other pulmonary conditions like COPD. The well-documented evolution of *Pa* during chronic infection to yield variants with mucoidy phenotype and altered QS and biofilm response could dramatically affect the dynamic interplay with other species like *Mab*. Understanding *Mab* coexistence with *Pa* phenotypes evolved during CF infection will offer novel insights for strain-specific interventions. Our future work will focus on investigating adaptation of *Mab* with *Pa* clinical strains from early and late stages of CF infection. Our discovery of a LasR-dependent bactericidal antagonist of *Mab* would suggest this loss of LasR signaling in *Pa* at late stages of infections (Bianconi et al., 2011; Lore et al., 2012; Baldan et al., 2014) may give *Mab* the upper hand. Finally, the high-level drug resistance and distinct drug susceptibility profiles of *Pa* and *Mab* already make treatment of polymicrobial infections challenging. The *Pa-*induced persister-like phenotype of *Mab* reflected in transcriptional signatures raises the possibility that interspecies interactions may impact antibiotic tolerance and treatment efficacy.

## Data Availability

Sequencing data generated in this study have been deposited in the NCBI’s SRA database and can be accessed under BioProject ID PRJNA1154599. All other relevant data are within the manuscript and in **Supplementary Material**.
