# Supplementary material for: Mycobacterium abscessus persistence in the face of Pseudomonas aeruginosa antagonism
Source: Front Cell Infect Microbiol. 2025 May 9;15:1569331. doi: 10.3389/fcimb.2025.1569331 (PMC12098619; doi:10.3389/fcimb.2025.1569331)
Supplement: Supplementary file 1 [file DataSheet1.docx]

Supplementary Material

# Supplementary Figures and Tables

## Supplementary Figures

**Supplementary Figure 1.** ***Mab growth in nutrient depleted media.*** Growth (CFU/ml) of *Mab* strains when grown in LB-Tween and in 50% diluted LB-Tween culture media at indicated times using the co-culture model conditions

**
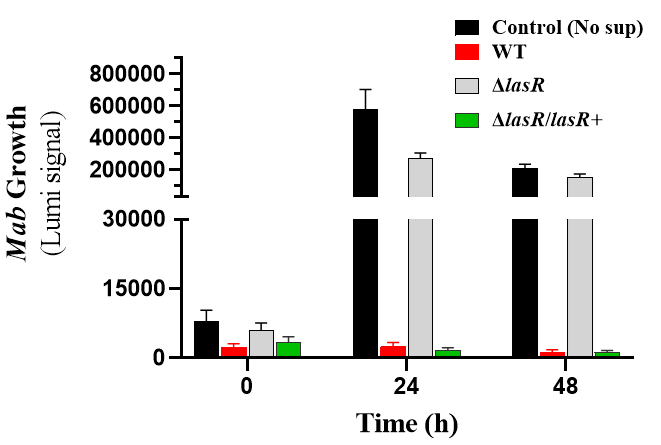
**

**Supplementary Figure 2.** ***lasR complementation***. *Mab* growth measured by luminescence signal after complementation of *lasR* mutation. The assay was carried out in 96-well microtiter plates with 50% v/v spent supernatants of *Pa* strains. The data is an average from 3 experiments each with a total of at least 9 technical replicates.

**
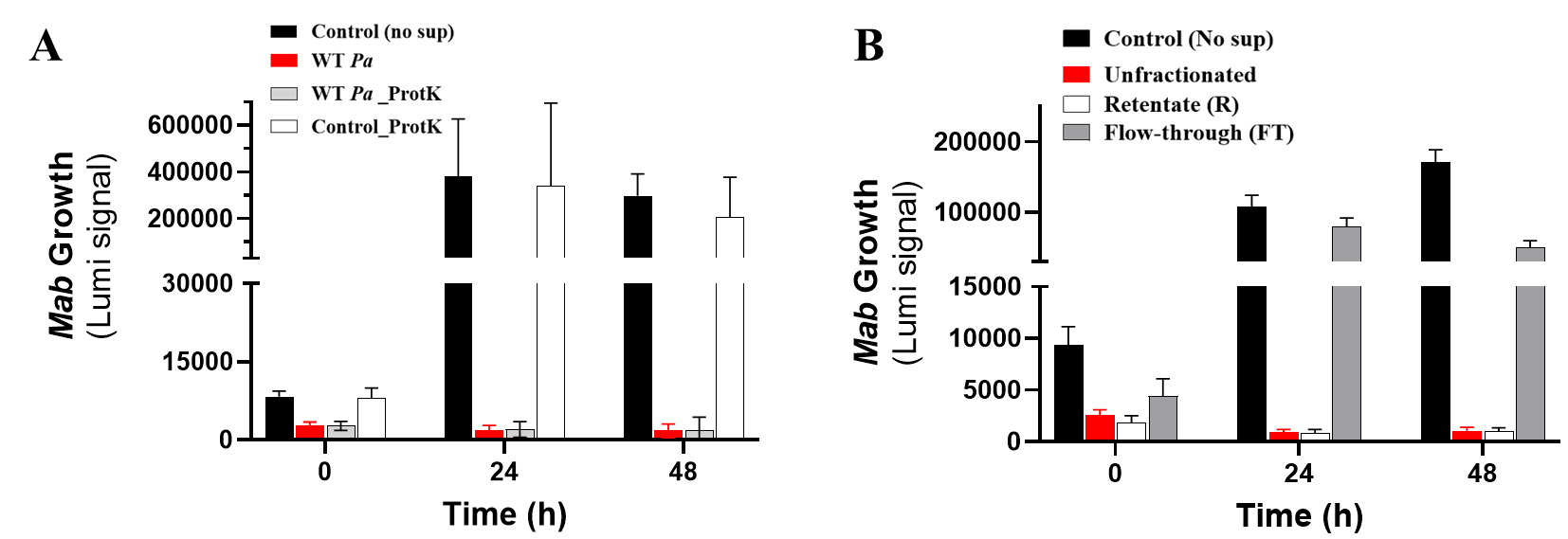
**

**Supplementary Figure 3.** *Mab-lux* growth in *Pa* WT supernatants (50% v/v) after **(A)** proteinase K (Prot K) treatment **(B)** Size fractionation (3kDa MWCO).

**
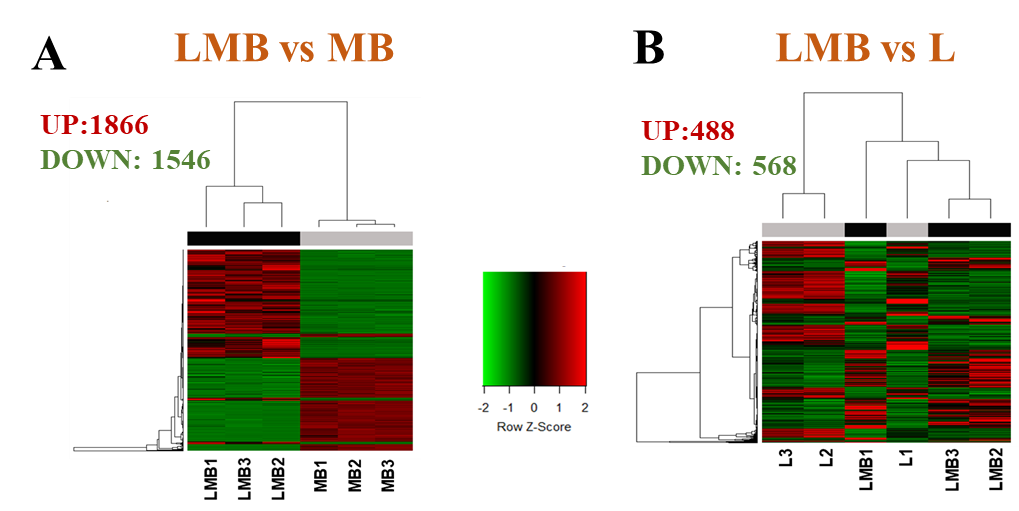
**

**Supplementary Figure 4:** Transcriptomic responses of *lasR* *Pa.* Heat maps showing differentially expressed genes of *Mab* (A) and *lasR* *Pa* (B) normalized counts per million of three replicates. L: ∆*lasR*, MB: *Mab*, LMB: ∆ *lasR* -*Mab* co-culture.

**
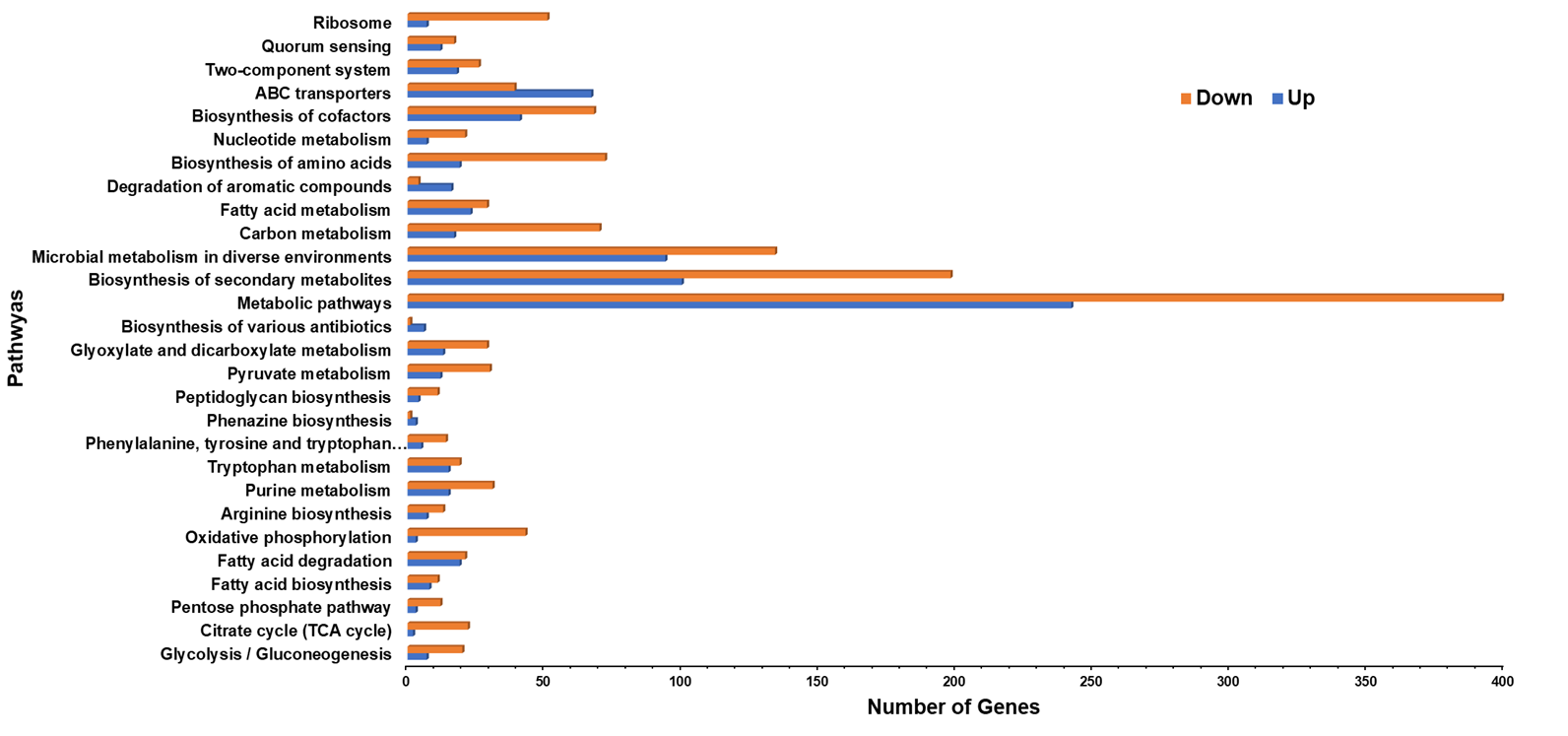
Supplementary Figure 5. Functional classification of *Mab* DEGs in a co-culture with WT *Pa* vs monoculture.** A bar chart showing select affected functional classes with up and down regulated (log_2_ ≥ 1 and FDR ≤ 0.05) genes.

## Supplementary Tables

**Supplementary Table 1.** *Pseudomonas aeruginosa* and *Mycobacterium abscesssus* specific oligonucleotide probes used for rRNA depletion

**Supplementary Table 2.** List of differentially expressed genes (DEGs) in a co-culture versus corresponding monoculture (log_2_FC -1 ≥1 to ≤1, p<0.05).

**Supplementary Table 3.** Differentially regulated *Mab* genes related to slow growth when partnered with *Pa*

**Supplementary Table 4.** Differentially regulated, horizontally acquired *Mab* genes in a co-culture with *Pa*

**Supplementary Table 5.** Differentially expressed carbon metabolism genes in *Mab*

**Supplementary Table 6.** Differentially expressed iron-responsive *Mab* genes in a co-culture with *Pa*

**Supplementary Table 7.** Virulence related *Mab* DEGs in a co-culture with *Pa*

**Supplementary Table 8.** Differentially expressed mono/dioxygenases *Mab* genes in a co-culture with *Pa*
